# Incremental Diagnostic Performance of Combined Parameters in the Detection of Severe Coronary Artery Disease Using Exercise Gated Myocardial Perfusion Imaging

**DOI:** 10.1371/journal.pone.0134485

**Published:** 2015-07-31

**Authors:** Chia-Ju Liu, Yen-Wen Wu, Kuan-Yin Ko, Yi-Chieh Chen, Mei-Fang Cheng, Ruoh-Fang Yen, Kai-Yuan Tzen

**Affiliations:** 1 Department of Nuclear Medicine, National Taiwan University Hospital, Taipei, Taiwan; 2 Division of Cardiology, Department of Internal Medicine, National Taiwan University Hospital, Taipei, Taiwan; 3 Department of Nuclear Medicine, Far-Eastern Memorial Hospital, New Taipei City, Taiwan; 4 Cardiology Division of Cardiovascular Medical Center, Far-Eastern Memorial Hospital, New Taipei City, Taiwan; 5 National Yang-Ming University School of Medicine, Taipei, Taiwan; University Francisco de Vitoria School of Medicine, SPAIN

## Abstract

**Purpose:**

Myocardial perfusion imaging (MPI) using gated single-photon emission tomography (gSPECT) may underestimate the severity of coronary artery disease (CAD). This study aimed to evaluate the significance of combined parameters derived from gSPECT, as well as treadmill stress test parameters, in the detection of severe CAD.

**Methods:**

A total of 211 consecutive patients referred for exercise MPI between June 2011 and June 2013 (who received invasive coronary angiography within six months after MPI) were retrospectively reviewed. Exercise MPI was performed with Bruce protocol and ^201^Tl injected at peak exercise. Gated SPECT was performed using a cadmium-zinc-telluride camera and processed by QPS/QGS software. Perfusion defect abnormalities such as sum stress score (SSS); sum difference score, algorithm-derived total perfusion deficits, transient ischemic dilatation ratios of end-diastolic volumes and end-systolic volumes, post-stress changes in ejection fraction, and lung/heart ratio (LHR) were calculated. Treadmill parameters, including ST depression (STD) at the 1st and 3rd minutes of recovery stage (1’STD and 3’STD), maximal STD corrected by heart rate increment (ST/HR), heart rate decline in 1^st^ and 3^rd^ minutes of recovery stage, recovery heart rate ratio (HR ratio), systolic and mean blood pressure ratios (SBP ratio and MAP ratio) during recovery phase were recorded. Diagnostic performances of these parameters were analyzed with receiver operating characteristic (ROC) analysis and logistic regression for detection of left main (≥ 50%) or 3-vessel disease (all ≥ 70% luminal stenosis) on invasive angiography.

**Results:**

Among various MPI and treadmill parameters used for detection of severe CAD, SSS and ST/HR had the highest AUC (0.78, 0.73, *p* = NS) and best cut-off values (SSS > 6, ST/HR > 17.39 10^-2^mV/bpm), respectively. By univariate logistic regression, all parameters except 1’HRR, 3’HRR, SBP and MAP ratios increased the odds ratio of severe CAD. Only increased L/H ratio, 3’STD, and HR ratio remained significant after multivariate regression. The predicted values of combined MPI and treadmill parameters (LHR, 3’STD, and HR ratio) gave the best ROC (AUC: 0.91) than any individual parameter or parameter combination.

**Conclusions:**

Of all treadmill and gSPECT parameters, the combination of MPI and treadmill parameters can offer better diagnostic performance for severe CAD.

## Introduction

Myocardial perfusion imaging (MPI) using gated single-photon emission tomography (gSPECT) is a useful imaging modality for the detection and risk stratification of coronary artery disease (CAD). However, it is well known that MPI may underestimate the severity and extent of CAD due to its relative quantification of perfusion defects, particularly in cases of balanced ischemia [1]. Although the problem of balanced ischemia can be solved by calculating coronary flow reserve using dynamic positron emission tomography [2, 3] or SPECT [4], additional costs and/or an acquisition algorithm are needed.

In addition to perfusion deficits, several stress-induced abnormalities [such as transient ischemic dilation (TID) and post-stress ejection fraction (EF)] can be derived from gSPECT [5–8], and increased pulmonary uptake. These stress-induced abnormalities have shown diagnostic and prognostic importance in patients with suspected CAD [9–11]. Left ventricular TID ratio is known as a functional parameter which could increase the sensitivity of MPI for severe CAD [12–16] and may be a specific prognostic marker for cardiovascular events [17–19]. Post-stress left ventricular stunning, in cases of decreased EF, is also an indicator of poor prognosis on gSPECT [18].

In addition, previous studies have shown that a number of variables obtained from the treadmill exercise test (TET) alone can be used to estimate prognosis in patients with suspected CAD. ST depression (STD) during the recovery stage [20], STD corrected by heart rate (HR) [21, 22], and post-exercise hemodynamic abnormalities, such as post-stress systolic blood pressure (SBP) [23–27] and HR changes during the recovery stage [28–31], are associated with higher risk of cardiovascular events and mortality.

In this retrospective study, the diagnostic performance of combined parameters was compared with gSPECT and TET parameters. The aim of this study was to evaluate the significance of combined parameters derived from gSPECT using novel cadmium-zinc-telluride (CZT) detectors, as well as treadmill stress test parameters, in the detection of severe CAD.

## Methods

Patients referred for exercise MPI between June 2011 and June 2013, who received invasive coronary angiography (CAG) within six months, were retrospectively reviewed. Patients were excluded if they had a history of myocardial infarction (MI), coronary artery bypass grafting, percutaneous coronary intervention, or documented congenital heart disease or severe valvular disease. The medical records (including demographics, cardiac risk factors, and medication) were reviewed for each patient. The pretest probability of CAD was evaluated using age, sex and angina typicality-based approach [32].

### Ethical statement

This study was approved by the institutional review board of National Taiwan University Hospital. Patients’ written informed consent was waived due to the retrospective nature of the study.

### Treadmill MPI protocol

MPI gSPECT was performed using the one-day exercise stress-rest protocol with injection of 3 mCi (111 MBq) thallium-201 (^201^Tl). Treadmill exercise stress was applied using the standard Bruce protocol with a 4 minute recovery stage. Endpoints of treadmill stress included achievement of 85% of age-adjusted maximal heart rate, >2 mm ST depression, systolic blood pressure (SBP) >250 mmHg, typical angina, frequent ventricular ectopy, hemodynamically compromised arrhythmia, or physical limitation.

Imaging acquisition was performed using the Discovery NM530c SPECT gamma camera (GE Healthcare, Haifa, Israel), which was equipped with solid-state CZT detectors. Projections were recorded on 32 × 32 pixelated (2.46 × 2.46 mm^2^) CZT elements. Maximum likelihood expectation maximization was used with a reconstructed voxel size of 4.0 × 4.0 x 4.0 mm^2^. A Butterworth post-processing filter was applied (order 10, cut-off frequency, 0.37) to the reconstructed slices [33].

### Treadmill parameters

Maximal ST depression (STD) and ST depression corrected by maximum HR (ST/HR) [21, 22] were recorded. STDs at 1 min and 3 min of the recovery stage (1’STD, 3’STD) were also recorded. The SBP and HR dynamic changes during recovery stage were reviewed as post-stress hemodynamic changes. SBP at 3 min of recovery stage was divided by the SBP at 1 min of recovery stage to manifest the decline in SBP from target exercise level and was recorded as the SBP ratio [34]. The difference between heart rate at peak exercise and that at 1st min and 3rd min of recovery stage were also recorded (1’HRR and 3’HRR, respectively) [8, 28]. HR recovery ratio (HR ratio) was calculated as ratio of HR at 3rd min of recovery stage and that at 1st min of recovery stage.

### MPI parameters

Semiquantitative interpretation of 17 segments from the short axis, vertical long axis, and horizontal long axis of each gSPECT was performed by two experienced nuclear physicians. Each segment was visually scored using a 5-point scale: 0 = normal, 1 = equivocal or mild, 2 = moderate, 3 = severe reduction of radiotracer uptake, and 4 = absence of radiotracer uptake. The scores of all 17 segments for both stress and rest images were summed to produce summed stress scores (SSS) and summed rest scores (SRS), respectively. The difference between SSS and SRS was recorded as the summed difference score (SDS). For gSPECT functional parameters, automated algorithm quantitative perfusion SPECT, and quantitative gated SPECT (QPS/QGS, Cedars-Sinai Medical Center, LA, USA) were used. Patients were excluded for functional parameter analysis if significant arrhythmia compromised the ECG gating.

The extent and severity of pixel-based cardiac perfusion defects at rest (rTPD) and post-stress (sTPD) were also compared and expressed as continuous parameters of QPS/QGS [35]. The difference between sTPD and rTPD was recorded as dTPD. The left ventricular end-diastolic volume (EDV) and end-systolic volume (ESV) and the transient dilatation ratios (TIDs) of EDV and ESV were calculated at stress and rest. TID ratios derived from hearts less than 20 ml (i.e., small hearts) were excluded from analysis. Both stress and rest left ventricular EF ratios (EF ratio) and differences (EF change), retrieved from the QGS algorithm, were calculated as manifestations of post-stress EF changes.

The same 3 × 3 cm square region of interest (ROI) was placed on the anterior wall and lung region above the anterior wall at a distance equal to two ROIs using the anterior view of maximal intensity projection (MIP). Placement of ROIs on areas with hypoperfused myocardium was avoided. The ratio of total radioactivity recorded from the ROIs of lung and myocardium were calculated as the lung/heart ratio (LHR). The post-stress LHR (sLHR) and increment in LHR (dLHR) from rest to stress were used as parameters of increased pulmonary uptake after stress.

### Coronary angiography results

CAG results performed within 6 months served as the gold standard. CAD was defined as more than 70% stenosis in any vessel. Severe CAD was defined as (1) left main arterial stenosis more than 50%, (2) three vessel disease with luminal stenosis of each major epicardial coronary artery more than 70%, and (3) two vessel disease including left anterior descending artery (LAD) with stenosis more than 70%.

### Statistical analysis

Continuous variables of patient groups were compared using the student *t*-test. Mann-Whitney test was used if normalcy of variables was rejected. Categorical variables were compared used Chi-square test. A *p* value < 0.05 was considered statistically significant. The sensitivity, specificity, positive predictive value (PPV), negative predictive value (NPV), and accuracy of each parameter were calculated and compared. The receiver operating characteristic (ROC) curve was used to compare the diagnostic performance of parameters and to determine cut-off values for identification of severe CAD, primarily using Youden index (except for the TID ratio). The cut-off values of TID ratio were determined using the mean value + 2 standard deviations (SDs) in patients without stenotic coronary arteries, according to CAG results [12, 13]. The cut-off criteria were also calculated to determine diagnostic performance using a combination of at least two different parameters, all of which had to be fulfilled simultaneously. The performance between cut-off values was compared using *McNemar* test or *Cochran’s Q* test.

## Results

A total of 211 patients were included for analysis. Most patients included had intermediate to high pretest cardiovascular risk and MPI was performed for risk stratification. Two hundred and five patients (97%) had interpretable ECG. Four patients (1.9%) with left bundle branch block and another two patients (0.9%) with ventricular pacing rhythms were excluded for ST-T change interpretation. MPI results of five patients (2.4%) with atrial fibrillation were still included since no rapid ventricular response was noted on baseline ECG. The exercise duration of these five patients all reached stage 2 of Bruce protocol with average METS of 4.6. Other abnormal rhythm noted on baseline ECG included eighteen patients (8.5%) with right bundle branch block, eight patients (3.8%) with first degree atrioventricular block, and six patients (2.8%) with left ventricular hypertrophy. Fifty-two patients (24.6%) had positive treadmill stress test indicative myocardium ischemia.

Most patients had intermediate to high pretest CAD risk (high risk: 61 patients; intermediate risk: 146 patients; and low risk: 4 patients). Among all patients, 35 (20%) patients were diagnosed with severe CAD. The demographic comparisons between patients with and without severe CAD are listed in Table 1. Patients with severe CAD had a higher prevalence of diabetes mellitus and hyperlipidemia, while no difference had been noticed regarding prescribed medication. Significant differences between the two groups were observed for SSS, SDS, TPD, EDV TID, dLHR, ST/HR, and recovery STD.

**Table 1:** The comparison of demographic data between patient with and without severe CAD.

	No severe CAD	Severe CAD	P value
Patients No.	176	35	<0.01
Age	59±10	59±9	0.98
BMI	26.4±3.7	25.5±3.2	0.21
METS	7.2±1.5	6.8±1.5	0.09
HTN	125 (71%)	25 (71%)	0.88
DM	44 (25%)	17 (49%)	0.01
Hyperlipidemia	98 (57%)	27 (77%)	0.02
Smoking	62 (35%)	13 (37%)	0.98
Medication			
Aspirin/Clopidogrel	87 (49%)	21 (60%)	0.34
Nitrate	33 (19%)	12 (34%)	0.07
Beta-blocker	71 (40%)	14 (40%)	0.88
ACEI/ARB	68 (39%)	17 (49%)	0.37
Statin/Fibrate	35 (20%)	7 (20%)	0.83
MPI parameters			
SSS	5.2±5.4	12.6±10.1	<0.01
SDS	4.1±4.0	8.3±5.4	<0.01
sTPD	8.5±7.6	16.1±11.6	<0.01
dTPD	4.2±6.4	9.0±6.6	<0.01
ESV TID	0.97±0.20	1.09±0.27	0.06
EDV TID	1.0±0.11	1.09±0.14	<0.01
EF ratio	1.05±0.15	0.97±0.19	0.17
EF changes	1.9±6.7	-2.8±13.2	0.10
sLH ratio	0.30±0.06	0.33±0.08	0.13
rLH ratio	0.32±0.05	0.32±0.06	0.70
dLH ratio	-0.02±0.05	0.01±0.05	<0.01
Treadmill parameters			
ST/HR	17.28±14.97	29.72±18.85	<0.01
1’ STD	0.34±0.53	0.78±0.72	<0.01
3’ STD	0.40±0.50	0.92±0.73	<0.01
SBP ratio	0.92±0.29	0.99±0.28	0.01
MAP ratio	0.98±0.17	1.01±0.23	0.07
1’HRR	23.1±9.8	24.3±10.4	0.05
3’HRR	43.7±12.0	50.4±10.3	0.10
HR ratio	0.81±0.12	0.86±0.07	0.01

Abbreviation:

BMI, body mass index; METS, metabolic equivalents; HTN, hypertension; DM, diabetes mellitus; ACEI, angiotensin converting enzyme inhibitors; ARB, angiotensin receptor blocker; rLH ratio, the lung/heart ratio at rest.

**Fig 1:**
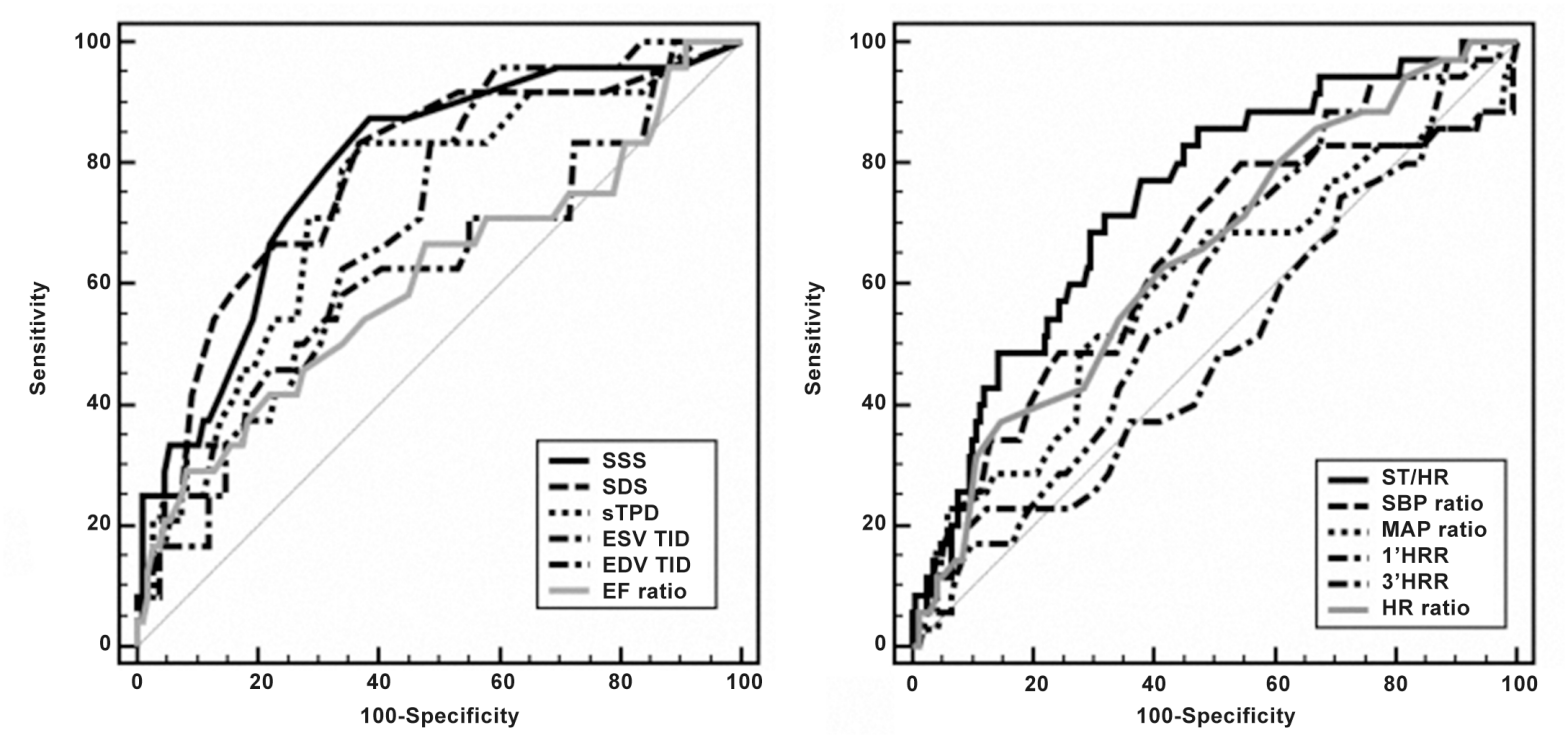
The receiver operating curves of myocardial perfusion imaging (MPI) and treadmill parameters in detection of severe CAD. The receiver operating curves of diagnostic performance of myocardial perfusion imaging (MPI) and treadmill parameters in detection of severe CAD were displayed on right and left panels. Summed stress score (SSS) and maximal ST depression corrected by maximum heart rate change (ST/HR) have highest area under the curve (AUC) among and treadmill parameters, respectively (AUC = 0.781 and 0.730).

The ROC analysis of TET and MPI parameters are displayed in Fig 1 and Table 2. SSS and ST/HR had the highest AUC among MPI and TET parameters (AUC = 0.781 and 0.730, respectively). The mean values of EDV TID and ESV TID in patients with patent coronary arteries on CAG were 0.99 ± 0.10 and 0.93 ± 0.17, respectively. The cut-off values of EDV TID and ESV TID were, therefore, set as 1.19 and 1.27, respectively, i.e., 2 SD above the mean values. Cut-off values of parameters other than TID are listed in Table 3. Corresponding sensitivity, specificity, PPV, NPV and accuracy of each TET, MPI and combined parameters are also listed in Table 3.

**Table 2:** The ROC analysis of MPI and TET parameters.

	AUC	SE	95% CI	P
SSS	0.781	0.0436	0.720 to 0.835	<0.01
SDS	0.763	0.0450	0.700 to 0.819	<0.01
sTPD	0.731	0.0458	0.666 to 0.789	<0.01
dTPD	0.727	0.0456	0.662 to 0.786	<0.01
EDV TID	0.707	0.0526	0.622 to 0.782	<0.01
ESV TID	0.621	0.0670	0.532 to 0.703	0.07
EF ratio	0.608	0.0696	0.520 to 0.691	0.12
EF change	0.604	0.0691	0.516 to 0.688	0.13
dLHR	0.673	0.0492	0.605 to 0.736	<0.01
ST/HR	0.730	0.0441	0.665 to 0.789	<0.01
1’STD	0.706	0.0500	0.639 to 0.767	<0.01
3’STD	0.717	0.0532	0.649 to 0.778	<0.01
1’HRR	0.512	0.0549	0.443 to 0.581	0.83
3’HRR	0.590	0.0487	0.519 to 0.657	0.07
HR ratio	0.650	0.0500	0.582 to 0.715	<0.01
SBP ratio	0.637	0.0566	0.568 to 0.703	0.02
MAP ratio	0.596	0.0576	0.526 to 0.664	0.09

**Table 3:** The cutoff vales of MPI, TET and combined parameters and corresponding diagnostic performance.

Treadmill parameters	Cut off values	Sensitivity	Specificity	PPV	NPV	Accuracy
STD	>1*	46	80	31	88	74
ST/HR	>17.39	77	61	28	93	63
3’ STD	>0.6	63	79	39	91	76
SBP ratio	>0.9	79	43	22	91	49
MAP ratio	>1.03	49	72	26	87	68
3HRR	<50	83	32	20	90	40
HR ratio	>0.87	37	83	30	97	75
STHR + SBP		60	77	35	91	75
STHR + 3HRR		66	73	33	91	72
STHR + SBP ratio + 3HRR		54	85	43	90	80
MPI parameters	Cut off values	Sensitivity	Specificity	PPV	NPV	Accuracy
SSS	>6	77	74	38	94	75
SDS	>4	80	66	32	94	68
sTPD	>8	80	63	30	94	65
dTPD	>5	71	64	28	92	65
EDV TID	>1.19	20	91	32	85	80
ESV TID	>1.27	20	86	23	84	75
EF ratio	<1.04	68	41	18	86	45
EF change	<-3	34	76	22	85	69
sLHR	>0.37	32	90	38	87	80
dLHR	>-0.04	88	37	21	94	45
SSS + EDV TID		20	97	54	86	84
SSS + EF ratio		54	80	35	90	76
SSS + dLHR		68	83	43	93	80
SSS + sLHR		24	97	57	87	85
MPI + Treadmill parameters		Sensitivity	Specificity	PPV	NPV	Accuracy
SSS + ST/HR		63	86	47	92	82
SSS + SBP		66	84	46	92	81
SSS + 3HRR		66	82	43	92	79
SSS + EDV + ST/HR		20	97	58	86	84
SSS + ST/HR + SBP		51	91	53	90	84
SSS + ST/HR + 3HRR		51	90	51	90	84
SSS + dLH + ST/HR		56	89	49	91	83
Logistic predict values		87	90	67	97	89

*>1mm with horizontal or downsloping ST depression, >2mm with upsloping ST depression in consecutive multiple leads.

Among MPI and treadmill parameters, SSS and ST/HR had the highest AUCs (0.78 and 0.73, respectively) and best cut-off values (SSS > 6 and ST/HR > 17.39 10^-2^mV/bpm, respectively) in detection of severe CAD. SSS, as well as other perfusion deficit scores (SDS, sTPD, and dTPD) had fair sensitivity of up to 80%. EDV and ESV TID had extremely low sensitivity (both 20%) but high specificity (91%, 86%, respectively) for severe CAD.

Criteria using combined cut-off values of SSS or ST/HR, in addition to one or two other parameters, are shown in Table 3. These criteria had high specificity and accuracy in the detection of severe CAD, but sensitivity was inevitably compromised.

Upon univariate logistic regression (Table 4), all parameters significantly increased the odds ratio of severe CAD except 1’HRR, 3’HRR, and mean arterial pressure ratios in recovery stage (MAP ratio). However, only dLHR, 3’STD, and HR ratio remained significant on multivariate logistic regression (Table 5). The predicted probabilities generated from multivariate logistic regression had the highest AUC (0.91) and best detection of severe CAD (sensitivity 87%, specificity 90%, PPV 67%, NPV 97%, and accuracy 89%) of any other individual or combination of parameters mentioned above (p < 0.01).

**Table 4:** The univariate logistic regression analysis.

	Odds ratio	95% CI	P
SSS	1.1361	1.0766 to 1.1989	<0.01
SDS	1.1873	1.1004 to 1.2810	<0.01
sTPD	1.0855	1.0441 to 1.1284	<0.01
dTPD	1.0997	1.0440 to 1.1583	<0.01
EDV TID*	1.0603	1.0224 to 1.0995	<0.01
ESV TID*	1.0255	1.0060 to 1.0454	0.01
EF ratio	0.0297	0.0012 to 0.7579	0.03
EF change	0.9389	0.8852 to 0.9958	0.04
dLHR*	1.1548	1.0631 to 1.2544	<0.01
ST/HR	1.0400	1.0189 to 1.0614	<0.01
1’STD	2.8165	1.6280 to 4.8728	<0.01
3’STD	3.7164	2.0303 to 6.8028	<0.01
1’HRR	1.0122	0.9758 to 1.0500	0.52
3’HRR	0.9770	0.9478 to 1.0072	0.13
HR ratio*	1.0531	1.0048 to 1.1036	0.03
SBP ratio*	1.0235	1.0037 to 1.0436	0.02
MAP ratio*	1.0274	0.9996 to 1.0560	0.05

* Multiplied by 100 for unit correction.

**Table 5:** Multivariate logistic regression of MPI TET parameters.

Variable	Coefficient	Std. Error	P
SSS	0.058750	0.038571	0.13
EDV TID*	0.056826	0.036858	0.12
EF_ratio	1.79116	2.29137	0.43
dLHR*	0.26390	0.088108	<0.01
ST/HR	-0.010108	0.043857	0.82
3’STD	2.21776	0.94335	0.02
HR ratio*	0.27037	0.083471	<0.01
SBP ratio*	0.0083895	0.021928	0.70
Constant	-34.4571		

*Multiplied by 100 for unit correction.

## Discussion

The identification of severe CAD based on abnormal MPI is important for referral physicians since management is critical in these patients. MPI is a useful imaging modality for the diagnosis of CAD. However, its limitation in severe extensive CAD, the so-called balanced ischemia, has been well documented. The perfusion defects visualized on MPI may underestimate the extent of coronary artery stenosis due to the lack of quantification of absolute coronary blood flow. Even using SSS > 6 as the cut-off, below 8, the sensitivity was still only 77%.

Severe functional parameters on gSPECT MPI are associated with severe CAD. Prior studies have found that TID could be used to diagnose severe CAD in conjunction with perfusion defects. In the present study, the cut-off values for TID were similar to prior investigations [12, 13, 36], however, the sensitivity (20%) was lower than expected. Prior studies had suggested the superior performance of ESV TID over EDV TID [37], while only EDV TID remained significant in this study. Possible explanations for this discrepancy include differences in radioisotopes used (^99m^Tc tracers vs. ^201^Tl), differences in stressors used (pharmacological vs. exercise), different imaging protocols, cameras, algorithms, methods of ratio calculation, or even differences in patients’ underlying disease such as diabetes or left ventricular hypertrophy [38, 39]. The cut-off values of normalcy and the sensitivity of TID for severe CAD also varied in prior studies [13–16]. The significantly high prevalence of small heart size (79 patients, 37%) may have contributed to the differences in TID results. Small LV size is not uncommon in the Asian population, especially in women [40–42]. Other functional parameters, such as decreased EF or increased pulmonary uptake after stress, are associated with worse prognosis [43].

Recently introduced SPECT using solid state CZT cameras has shown better intrinsic performance compare with Anger cameras [44, 45]. The significant reduction in acquisition time allows for rapid imaging before recovery of post-stress stunning [46]. However, the differences in prognostic value between gated functional parameters derived from different camera systems have not yet be validated. Although ventricular volume and EF obtained from CZT cameras and Anger cameras have shown good agreement based on prior studies [47, 48], the cut-off values for post-stress abnormalities may be different. For example, the upper limit of post-stress lung/heart ratio (0.41) of patients without CAD in our study was apparently lower than that previously reported [49, 50]. Furthermore, gSPECT using ^201^Tl is clinically feasible using CZT cameras because of higher sensitivity [44, 45, 51], but it could still contribute to differences in functional parameters on gSPECT [52, 53].

The criteria of positive TET result using significant STD was not sensitive enough for the diagnosis of CAD. Using STD >2 mm for severe CAD had poor sensitivity (46%) in this study. However, the HR corrected STD or STD in the recovery stage showed better performance than the uncorrected STD. In addition, abnormal hemodynamic changes after exercise have well established prognostic relevance regarding cardiovascular events and mortality. The delayed decline of SBP during the recovery stage of TET, the so-called paradoxical SBP elevation, was shown to be associated with severe CAD in prior studies [23, 26, 34]. However, like MPI parameters, none of TET parameters rendered acceptable cut-off values.

The majority of patients included in the current study had intermediate and high pre-test cardiovascular risk. However, estimating pre-test probability of angiographically significant CAD with traditional age, sex, and angina typicality-based approach sometimes overestimates the actual prevalence of disease[54]. Therefore stress tests might provide incremental value of risk stratification. The possibility of severe CAD is increasing in patients with intermediate and high risk. The balanced ischemia on MPI might underestimate the disease severity, it is important to consider the risk factors, symptoms and prior treadmill exercise test results. The increment of diagnostic performance of combined MPI and treadmill parameters could be expected; however, previous studies mainly focused on validation of the diagnostic or prognostic significance of CAD of single test, and limited data validated the combination of treadmill ECG and MPI variables in patients with intermediate and high risk groups to our best knowledge. The majority (97%) of enrolled patients in this study had interpretable ECG and was feasible for analysis. The addition of TET to the gSPECT parameters offered better diagnostic accuracy than the individual test, primarily through an improvement in specificity. Using multivariate logistic regression, the probability of severe CAD could be predicted well by a combination of dLHR, 3’STD, and HR ratio. It is worth mentioning that LHR should be calculated despite the small field of view of the CZT camera considering its prognostic significance based on our results. Using the predicted probabilities as a cut-off had high sensitivity, specificity, and accuracy for diagnosis of severe CAD. For the few patients with non-evaluable ECG, as the other six patients (3%) in this study, the MPI parameters were still helpful with the acceptable accuracy.

The major limitations of this study included its retrospective nature and small sample size with heterogeneous characteristics. Only patients who underwent coronary angiography were included in the analysis which may have caused a selection bias. The frequency of coronary risk factors was high in the enrolled subjects, which might limit the generalization for clinical application.

In conclusion, although further prospective validation in a larger population is needed, our results encourage the wide use of treadmill as a stressor for MPI since these treadmill parameters could be easily retrieved from daily practice without additional cost or algorithm.
